# Fear of missing out and depressive symptoms during the COVID-19 pandemic

**DOI:** 10.1111/spc3.12828

**Published:** 2023-06-21

**Authors:** Angie S. LeRoy, Vincent D. Lai, Arya Tsay-Jones, Christopher P. Fagundes

**Affiliations:** 1 Department of Psychology & Neuroscience, Baylor University, Waco, Texas, USA; 2 Department of Psychological Sciences, Rice University, Houston, Texas, USA; 3 Department of Behavioral Sciences, The University of Texas MD Anderson Cancer Center, Houston, Texas, USA; 4 Department of Psychiatry & Behavioral Sciences, Baylor College of Medicine, Houston, Texas, USA

**Keywords:** fear of missing out, depressive symptoms, perceived stress, loneliness, COVID-19 pandemic, older adults, mental health

## Abstract

During the early stages of the COVID-19 pandemic, governments issued public health safety measures (e.g., “stay-at-home” ordinances), leaving many people “missing out” on integral social aspects of their own lives. The fear of missing out, popularly shortened as, “FoMO,” is a felt sense of unease one experiences when they perceive they may be missing out on rewarding and/or enjoyable experiences. Among 76 participants (ages *M* = 69.36, *SD* = 5.34), who were at risk for hospitalization or death if infected with COVID-19, we found that FoMO was associated with depressive symptoms at Time 1, even when controlling for perceived stress, loneliness, and fear of COVID-19. However, FoMO did not predict future depressive symptoms, about 1 week later, when controlling for Time 1 depressive symptoms. These findings provide further evidence that FoMO is associated with depressive symptoms in a short period of time even when accounting for other powerful social factors such as loneliness. Future research should explore the potential causal relationships between FoMO and depression, especially those that may establish temporal precedence.

## 1 | INTRODUCTION

In recent years, researchers have taken “FoMO,” from its place as a buzzword in popular vernacular to an established psychological phenomenon (Baker et al., 2016; Przybylski et al., 2013). The fear of missing out, or “FoMO,” is generally defined as a “pervasive apprehension that others might be having rewarding experiences from which one is absent” (Przybylski et al., 2013, p. 1). When the COVID-19 outbreak hit the U.S. in early 2020, governments issued “lockdown” and “social distancing” ordinances to prevent viral spread (Goolsbee et al., 2020). These unprecedented circumstances left most people quite literally “missing out” on integral social aspects of their own lives–from everyday social interactions (e.g., casual hangouts with friends), to important life milestones (e.g., graduation ceremonies, weddings).

Given the degree to which “missing out” on social situations had become a reality during the early stages of the pandemic (e.g., April-November 2020), we sought to determine whether FoMO would be associated with depressive symptoms among people who were most at risk for the negative health consequences associated with COVID-19 (e.g., hospitalization and/or death), as had been reported in pre-pandemic FoMO research (Baker et al., 2016; Kartol & Gündoğan, 2020; Yuan et al., 2021). We were particularly interested in people at-risk for COVID-19, as we suspected that they were more likely to follow social distancing mandates. Further, since people often experience FoMO while doing a *required* task (Milyavskaya et al., 2018), we thought FoMO may be particularly problematic for people during the early stages of the pandemic when people were regularly engaging in required behaviors (e.g., reduced social contact, mask-wearing, working from home). Specifically, we hypothesized that Time 1 levels of FoMO (i.e., participants’ reports of FoMO when entering the study) would be positively associated with Time 1 depressive symptoms (i.e., participants’ reports of depressive symptoms) over and above perceived stress, loneliness, and fear of COVID-19. We also explored whether Time 1 FoMO predicted later depressive symptoms about 1 week later (i.e., at follow-up).

## 2 | METHOD

### 2.1 | Participants

An a priori power analysis using G*Power indicated that a minimum of 63 participants was required in order to have 80% power for detecting a medium-sized effect when employing the traditional 0.05 criterion of statistical significance. Therefore, a subsample of 76 participants (21 male, 55 female) who were on average 69.36 (*SD* = 5.34, range = 58–81) years old, and at risk for the most severe negative health outcomes associated with COVID-19 (97% reported at least one underlying health condition) were obtained from a larger study examining whether journaling about different topics affects people’s health during the COVID-19 pandemic (LeRoy et al., 2023). The sample was predominantly White (88.2%), 5.3% Black/African American, 2.6% Asian, 2.6% Native Hawaiian/Pacific Islander, and 1.3% multi-ethnic participants. Additionally, the sample was 44.7% married, 21.1% single, 17.1% divorced or separated, 15.8% widowed; 1.3% were in a committed long-term relationship, but not living with their partner. Between April-November 2020, quarantine and social distancing policies were widely in place and vaccines for COVID-19 were not yet generally accessible (Katella, 2021). During this period, participants from across the United States were recruited through several means, including senior communities, churches, social media, and ResearchMatch.

### 2.2 | Procedure

Interested participants completed an online eligibility screening form via Qualtrics. Participants must have been able to read and write in English AND they must have been 60 years or older OR have an underlying medical condition (i.e., asthma, lung disease, heart conditions, being immunocompromised, obesity, diabetes, kidney disease, and/or liver disease). Participants over 60 did not need to have an underlying health condition to participate, as they were already at risk for more severe consequences of COVID-19 infection due to their age alone. Eligible participants accessed a consent form and completed Time 1 questionnaire measures. Then, participants were randomly assigned to one of three writing conditions (security prime, self-regulation, or control) and completed their associated writing prompts for 20 min per day over 3 days of 1 week, as part of the larger study. Participants completed follow-up questionnaires about a week after completing their Time 1 questionnaire, and about 48 h after completing their last writing session. The current study utilized the subsample of participants who were randomly assigned to the writing control condition in which they were asked to write objectively about their living spaces (e.g., kitchen, closet, bathroom; LeRoy et al., 2023).^ 1^ Finally, participants were routed to a debriefing sheet, and then were compensated with a $15 gift card via email and provided with a mental health resource sheet.

### 2.3 | Measures

#### 2.3.1 | Demographic variables & social media usage

Participants were asked to self-report their age, gender, relationship status, the number of people living in their household, and how many minutes they spent using social media per day.

#### 2.3.2 | Fear of missing out (FoMO)

Participants rated the degree to which they related to the following two items from the Fear of Missing Out scale, “I fear others have more rewarding experiences than me,” and “I get worried when I find out my friends are having fun without me,” (Przybylski et al., 2013). Responses were scored on a 5-point scale (1 = *not at all true of me*; 2 = *slightly true of me*; 3 = *moderately true of me*; 4 = *very true of me*; 5 = *extremely true of me*). To capture “FoMO,” we averaged participants’ scores on these items (*α* = 0.81), with higher scores indicating more FoMO, and lower scores indicating less FoMO. We selected only two items from the FoMOs to reduce participant burden, as participants were already completing several measures and activities as part of the larger study; as seen in other research, even brief FoMO measures (e.g., Hartanto et al., 2022; Riordan et al., 2018) can be effective in capturing participant’s general experiences of FoMO.

#### 2.3.3 | Perceived Stress Scale

Participants completed the four-item Perceived Stress Scale 4 (PSS-4; Cohen et al., 1983), which asks about the frequency of perceived stress (e.g., “In the last month, how often have you felt that you were unable to control the important things in your life?”), on a 5-point scale (0 = *never*; 1 = *almost never*; 2 = *sometimes*; 3 = *fairly often*; 4 = *very often*). We calculated a sum score of these four items (including reverse-scored items), which demonstrated good internal consistency (*α* = 0.86), with higher scores indicating greater perceived stress, and lower scores indicating lower perceived stress.

#### 2.3.4 | Loneliness

Participants completed the 3-item Loneliness Scale (developed from the Revised UCLA Loneliness Scale; Hughes et al., 2004). Each question corresponds with a loneliness dimension: relational connectedness, social connectedness, and self-perceived isolation. For example, participants responded to the item, “How often do you feel isolated from others?” on a 3-point scale (1 = *hardly ever*; 2 = *some of the time*; 3 = *often*). The three items showed good internal consistency (*α* = 0.85) and were summed to determine an overall loneliness score, with higher scores indicating more perceived loneliness, and lower scores indicating less perceived loneliness.

#### 2.3.5 | The COVID-19 Worry Index

Participants completed the COVID-19 Worry Index (Rogers et al., 2021), a 15-item measure assessing worry about contracting COVID-19, and associated symptoms and health consequences. Respondents were asked to rate their worry about each item (e.g., “I worry that if I were to contract COVID-19, medical help would not be adequate in helping me with my symptoms,”) on a 1 (*Not at all*) to 7 (*A great deal*) scale. In an effort to parallel the study designs used by other FoMO researchers that have investigated fear of COVID-19 (Bayin et al., 2021) using similar measures (i.e., the Fear of COVID-19 Scale; Ahorsu et al., 2022), we intentionally selected seven items that were most similar to those found on the Fear of COVID-19 scale–particularly, those that reflect worry primarily about *oneself* contracting the virus, rather than others. Thus, the COVID-19 worry variable in the current study reflected a mean score of these seven items (*α* = 0.91), with higher scores indicating more fear of COVID-19.

#### 2.3.6 | Depressive symptoms

Depressive symptoms were assessed using the 8-item Patient-Reported Outcomes Measurement Information System (PROMIS) Depression bank, short form 8a (Cella et al., 2010). Participants responded to items such as, “In the past week…I felt worthless,” and “…I felt that nothing could cheer me up,” on a 5-point scale (1 = *never*; 2 = *rarely*; 3 = *sometimes*; 4 = *often*; 5 = *always*). Item scores were averaged to determine an overall depressive symptoms score, such that higher scores indicated a higher frequency of depressive symptoms at Time 1 (*α* = 0.92) and follow-up (*α* = 0.92).

## 3 | RESULTS

Data were analyzed using primarily IBM SPSS Statistics version 28.0 (IBM Corp, 2021).^ 2^ Descriptive statistics and bivariate correlations between variables of interest are reported in Table 1. We selected potential covariates based on previous FoMO research and inspected whether these variables (e.g., age, gender; Elhai et al., 2018) were associated with depressive symptoms in the current study. To avoid overfitting the model, we only included variables as covariates (i.e., perceived stress, loneliness, fear of COVID-19) if they demonstrated a significant association (*p* < 0.05) with our outcome variable, depressive symptoms. Prior to conducting linear regression analyses, we examined assumptions of normality, linearity, and homoscedasticity, along with evaluating collinearity statistics (all VIFs <1.74).

A four-step hierarchical multiple regression (Table 2) revealed that, at Step 1, perceived stress accounted for 63% of the variability in depressive symptoms, *F*(1, 74) = 127.40, *p* < 0.001. At Step 2, introducing loneliness into the model explained an additional 5% of variability in depressive symptoms, *F*(1, 73) = 11.65, *p* = 0.001. At Step 3, while the overall model was significant, introducing fear of COVID-19 into the model did not uniquely explain any additional variation in depressive symptoms, *F*(1, 72) < 1, *p* = 0.991. However, at Step 4, introducing FoMO into the model explained an additional 3% of the variance, *F*(1, 71) = 7.28, *p* = 0.009.

To explore possible causal relationships between FoMO and depressive symptoms, we tested whether earlier feelings of FoMO (at Time 1) predicted depressive symptoms approximately 1 week later, and vice versa. To test these exploratory hypotheses, we conducted a two-step hierarchical multiple regression for each research question using follow-up data from 65 participants. We found that Time 1 FoMO did *not* predict depressive symptoms at follow-up, when controlling for Time 1 depressive symptoms (*p* = 0.338). Likewise, when controlling for Time 1 FoMO, Time 1 depressive symptoms failed to predict FoMO, a week later (*p* = 0.327).

## 4 | DISCUSSION

Among those most at risk for the adverse health consequences associated with COVID-19 (e.g., hospitalization and/or death), we found that FoMO at Time 1 was associated with depressive symptoms at Time 1, replicating the effects of previous pre-pandemic FoMO research reports (Baker et al., 2016; Kartol & Gündoğan, 2020; Yuan et al., 2021). Further, this relationship held even when controlling for perceived stress, loneliness, and fear of COVID-19. Time 1 FoMO did not, however, predict depressive symptoms about a week later, after controlling for Time 1 depressive symptoms. Likewise, Time 1 depressive symptoms did not predict FoMO reported about a week later, after controlling for earlier FoMO. FoMO may be a social/perceptual feeling that reflects and impacts mood states in the moment, but less so in the near future. However, our current study design did not allow for us to test for the effects of FoMO on depressive symptoms, or depressive symptoms on FoMO, in the more distant future. Further, FoMO and depressive symptoms could be caused by the same third variable, which remains unidentified. More research is needed to better understand how FoMO fluctuates across contexts and over time, and to better establish possible causal relationships between FoMO and depressive symptoms.

These data were collected as part of a larger study investigating whether an online security-priming writing intervention, which aimed to enhance feelings of safety and security during the pandemic, significantly reduced distress levels, compared to another writing intervention which aimed to facilitate self-regulation. We also included a comparison control group in which participants wrote objectively about household items during their writing sessions, which occurred for 20 min per day, for 3 days of 1 week, in between the Time 1 and follow-up measurements. We anticipated that the two experimental writing conditions would pose the biggest risk of reducing the possible increases in later depressive symptoms. Thus, only the subsample of participants who completed the control writing prompts were included in the analyses reported in the current manuscript. Nevertheless, it is possible that the writing activities participants engaged in as part of the larger study may have counteracted any possible increases in later depressive symptoms and limited the variability in depressive symptoms at the 1-week follow-up. Thus, we treat any conclusions drawn from our exploratory analyses on the follow-up data with caution.

At the onset of the pandemic, some questioned the relevance of studying FoMO during such a unique time, as social distancing measures restricted the time people spent with others, creating less rewarding social experiences and thus fewer opportunities for a person to compare their experiences with others’ (Casale & Flett, 2020). Nevertheless, during the pandemic, FoMO has been associated with greater loneliness and social networking use (Fumagalli et al., 2021), depression, and anxiety (Ibrahim et al., 2022), across age groups (Durao et al., 2023), further validating that FoMO is a real psychological problem associated with significant health outcomes.

Indeed, because the FoMO construct originated in the American English vernacular and was first studied primarily among college students and in the context of social media (young people are more frequently the users of social media; e.g., Chou et al., 2009), it may be easy for some to dismiss FoMO as a “young people problem.” As we searched through the current literature on FoMO, it was our observation that there are more studies post-COVID-19, which include an older demographic of research participants, compared to pre-pandemic FoMO studies, suggesting a paradigm shift in how researchers are thinking about FoMO. The current study findings further demonstrate the importance of including people from higher age groups in FoMO studies, especially those studies conducted post-pandemic.

Overall, our study is unique in that it further establishes FoMO as an important associate with depressive symptoms, particularly in a pandemic context. However, the causal relationship between these two variables (e.g., which precedes the other) remains unclear. FoMO may be associated with depressive symptoms in ways that are unique to other social/perceptive stress such as general perceived stress, loneliness, and a general fear of COVID-19. Future research should test potential causal relationships between FoMO and depressive symptoms to better establish temporal precedence.

## Figures and Tables

**TABLE 1:** Means, standard deviation, and bivariate correlations among study variables.

Variable	M or #	SD or *%*	1	2	3	4	5	6	7	8	9	10
1. FoMO	1.63	0.89	-									
2. Time 1 depressive symptoms	1.94	0.87	0.62**	-								
3. Follow-up depressive symptoms	1.64	0.77	0.58**	0.83**	-							
4. Perceived stress	4.79	3.37	0.51**	0.80**	0.74**	-						
5. Loneliness	5.50	2.01	0.56**	0.56**	0.60**	0.45**	-					
6. Fear of COVID-19	2.81	1.31	0.39**	0.34**	0.40**	0.40**	0.28*	-				
7. Age	69.36	5.34	−0.07	−0.07	−0.06	−0.16	0.03	−0.02	-			
8. Gender	55	72.4	0.09	0.07	−0.10	0.08	−0.02	−0.08	−0.25*	-		
9. No. of people in household	1.59	0.64	−0.16	−0.08	−0.04	−0.06	−0.23*	−0.01	−0.20	−0.17	-	
10. Social media use	56.63	74.79	0.01	0.09	−0.004	0.07	0.07	0.004	−0.155	0.09	0.16	-

*Note*: Gender coded 1 = female, 0 = male; # reflects number of females; social media use = minutes/day.

* *p* < 0.05,

** *p* < 0.01.

**TABLE 2:** Hierarchical multiple regression depicting depressive symptoms as a function of variables of interest at each step of analysis.

Predictor	*b*	*SE*	*β*	*p*	*R* ^2^_adj_	Δ*R*^2^_adj_
Step 1					0.63***	
Perceived stress	0.20***	0.02	0.80	<0.001		
Step 2					0.67***	0.05***
Loneliness	0.11**	0.03	0.25	0.001		
Step 3					0.67***	−0.00
Fear of COVID-19	0.001	0.05	0.001	0.991		
Step 4					0.70***	0.03**
FoMO	0.22**	0.08	0.23	0.009		

*Note*: A significant *b* indicates the *β* is also significant.

* *p* < 0.05,

** *p* < 0.01,

*** *p* < 0.001.

## Footnotes

1 The subsample of data reported here are those collected from only the participants in the control writing condition of the study. However, in the larger writing study, which included participants who completed writing prompts from three distinct writing conditions, there were no significant differences in depressive symptoms between any of the writing study conditions pre-to-post intervention (*p*s > 0.05). Further, the statistically significant results reported in the current article are not isolated to the subsample; results are the same whether we use the subsample or if we collapse across writing conditions and also include participants from the other writing conditions, creating 1 larger sample (Time 1 *N* = 195; follow-up *N* = 172).

2 Analyses were conducted twice (once by each first-author), using two different programs (SPSS & R).
